# Role of biophysical stimulation in multimodal management of vertebral compression fractures

**DOI:** 10.1016/j.csbj.2023.11.023

**Published:** 2023-11-14

**Authors:** Alberto Di Martino, Eleonora Villari, Riccardo Poluzzi, Matteo Brunello, Valentino Rossomando, Claudio D’Agostino, Federico Ruta, Cesare Faldini

**Affiliations:** a 1st Orthopaedic and Traumatologic Department, IRCCS Istituto Ortopedico Rizzoli, Via G.B. Pupilli 1, 40136 Bologna, Italy; b Department of Biomedical and Neuromotor Sciences, University of Bologna, Italy

**Keywords:** Vertebral compression fractures, Fragility fractures, Biophysical stimulation, Capacitive-coupling

## Abstract

Raised life expectancy and aging of the general population are associated with an increased concern for fragility fractures due to factors such as osteoporosis, reduced bone density, and an higher risk of falls. Among these, the most frequent are vertebral compression fractures (VCF), which can be clinically occult. Once the diagnosis is made, generally thorough antero-posterior and lateral views of the affected spine at the radiographs, a comprehensive workup to assess the presence of a metabolic bone disease or secondary causes of osteoporosis and bone frailty is required. Treatment uses a multimodal management consisting of a combination of brace, pain management, bone metabolism evaluation, osteoporosis medication and has recently incorporated biophysical stimulation, a noninvasive technique that uses induced electric stimulation to improve bone recovery through the direct and indirect upregulation of bone morphogenic proteins, stimulating bone formation and remodeling. It contributes to the effectiveness of the therapy, promoting accelerated healing, supporting the reduction of bed rest and pain medications, improving patients’ quality of life, and reducing the risk to undergo surgery in patients affected by VCFs. Therefore, the aim of this review is to outline the fundamental concepts of multimodal treatment for VCF, as well as the present function and significance of biophysical stimulation in the treatment of VCF patients.

## Graphical Abstract

**Figure:**
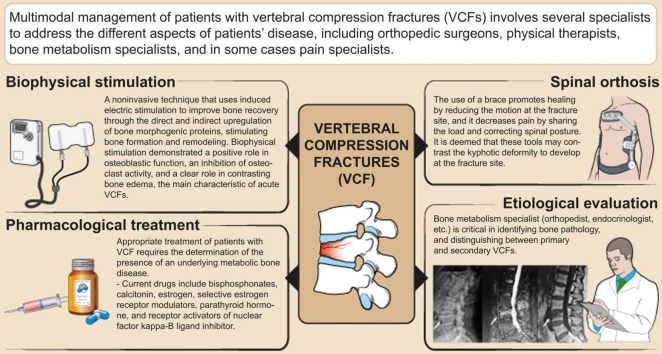

## 1 Introduction

Raised life expectancy and aging of the general population are associated with an increased risk of fractures. Among those typically occurring in the older adults, fragility fractures represent a rising concern for public health, usually occurring on a pathologically weakened bone [1]. Osteoporosis and reduction in bone density, together with the increase in fall events are typically observed in the older adults, because of the higher occurrence of sarcopenia, neuromuscular pathologies, or cognitive impairment [2]. In the United States, it was estimated that 54 million adults over the age of 50 have a decreased bone mass [3]. Among these, 40–50% of women over 50 years of age will encounter fragility fractures during their lifetime because of the intrinsic increased risk of osteoporosis and poor bone metabolism occurring after menopause [4], [5].

Among fragility fractures, the most frequent are vertebral compression fractures (VCF), which primarily occurs in the older adults as a result of a low-energy trauma in patients already suffering from osteoporosis, even though, in the case of severe osteoporosis, VCF can occur while doing something simple like coughing or sneezing [6].

Most VCF are clinically occult; in fact, about 1 in 3 vertebral fragility fractures are identified clinically, with only a small percentage requiring hospitalization [5]. Back pain is the main symptom, occurring in 85% of patients. A VCF is identified with a reduction in the height of the vertebral body by at least 15–20% and most commonly regards the thoracolumbar junction.

**Fig. 1:**
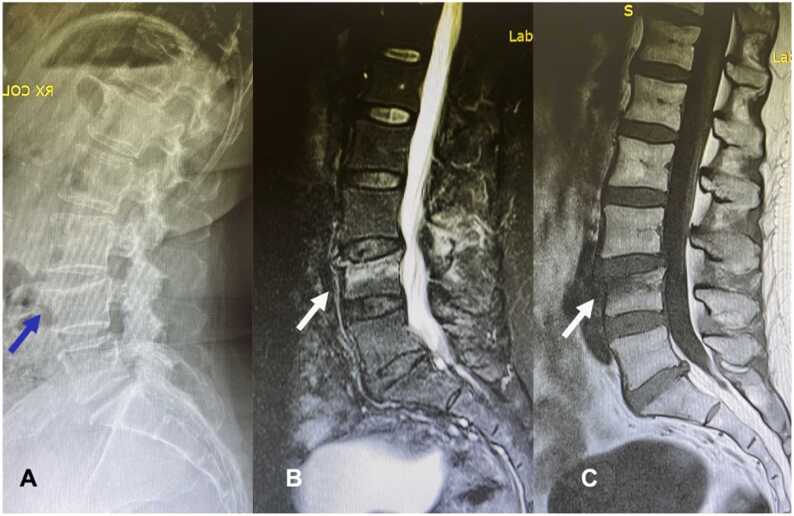
(A) VCF of L4; blue arrow shows the point of decreased height of the anterior portion of the vertebral body compared to the posterior wall; (B) T2- STIR sagittal and (C) T1 sagittal images confirm the presence of the bone edema (arrows) typical of the acute fractures.

Diagnostic imaging includes antero-posterior and lateral views of the affected spine at the radiographs. To meet radiographic criteria, vertebral body height should decrease by at least 20% on the anterior wall compared to the posterior wall, or by at least 4 millimeters from baseline height (Fig. 1-A) [7]. However, the most significant challenge is to distinguish between recent vs old fractures (timing of the fracture). Considering that recent fractures exhibit bone edema, magnetic resonance imaging (MRI) is the most useful tool to define the time of fracture, and can recognize pathologic fractures in case of metastatic or primary tumor involvement of the bone (Fig. 1-B,C). MRI is also a useful tool to monitor the healing of the fracture because bone edema regresses during the fracture healing.

**Fig. 2:**
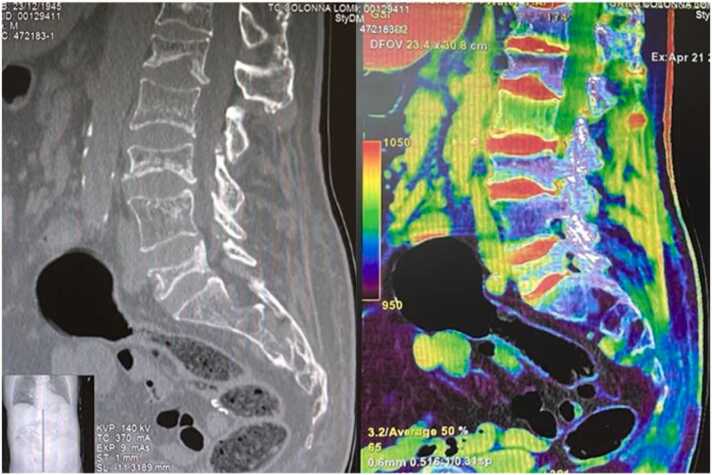
Comparison of the same patient complaining of low back pain and a history of multiple VCFs. On the left, the sagittal CT scan shows multiple VCFs, leaving doubts about which are the acute VCFs. On the right image acquired by the dual energy technique, vertebral bodies are colored in green when bone edema is present, as in acute fractures, and in blue in healed fractures. The picture outlines acute fractures of L2 and L5, and healed fractures of T12, L1, L3, and L4.

CT scan is an effective screening tool for vertebral fractures. Most recently, Dual Energy-CT (Fig. 2) has been proposed as an alternate imaging exam for the assessment of VCF because of its ability to outline the presence of bone edema, giving information about bone healing and time from fracture. Also, it can be performed also in patients with pacemakers or other implanted devices, in which the performance of MRI is contraindicated [8].

In a patient with the diagnosis of VCF a comprehensive workup to assess the presence of a metabolic bone disease or secondary causes of osteoporosis and bone frailty is required. Dual-energy x-ray absorptiometry at the lumbar spine and proximal femur is generally performed to check for osteoporosis and risk of fracture. Laboratory evaluation including a complete blood count; complete metabolic workout with kidney function; measurement of erythrocyte sedimentation rate and thyroid-stimulating hormone, 25-hydroxyvitamin D, parathyroid hormone, and C-reactive protein levels is assessed [5]. Once the diagnosis is made and the patient is evaluated for risk factors, treatment is planned, which at present requires an integrated envision and management of the different aspects of the patient’s bone health, both metabolic and biomechanics [9]. Multimodal therapy of VCF in several Western Countries, has recently incorporated Biophysical Stimulation.

The aim of this review is therefore to describe the main principles of multimodal treatment of VCF, outlining the current role and contribution of biophysical stimulation to the management of VCF patients. Current evidence from experimental in vitro and in vivo studies, and most recent clinical trials on the topic, will be categorized and reported.

## 2 Multimodal management of patients with VFC

**Fig. 3:**
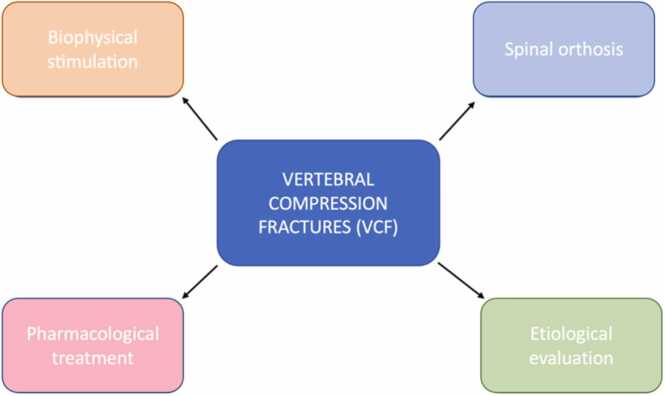
Multimodal management of VCF includes pharmacological treatment, spinal orthosis, etiological evaluation, and biophysical stimulation.

Multimodal management (MM) of patients with VCFs involves several specialists to address the different aspects of patients’ disease, including orthopedic surgeons, physical therapists, bone metabolism specialists, and in some cases pain specialists. The mainstays of current standards in MM of VCF include a combination of immobilization with a brace, pain management, exclusion of potential secondary forms of bone metabolic diseases, and prescription of osteoporosis medication (Fig. 3) [10]. Therapy is aimed at the promotion of the healing of fractures, and this may be supported by biophysical stimulation [11]. Patients failing conservative treatment often require surgery, which may be of cementoplasty, or a spinal instrumentation, performed by a standard or minimally invasive approach [12].

### 2.1 Etiological evaluation and pharmacological treatment

Appropriate treatment of patients with VCF requires the determination of the presence of an underlying metabolic bone disease. Therefore, a comprehensive workup, most of the time by blood and urine samples harvest analysis is the beginning of the management of the patient. In most patients, primary osteoporosis will be the principal cause of the VCF. However, at present time, secondary forms of osteoporosis include hypogonadism, endocrine disorders, gastrointestinal diseases, transplantation, genetic disorders, and use of medications, some of which are frequently encountered also in male patients. In the case of diagnosis of a secondary metabolic bone disease, targeted therapy is started [13]. One of the most common forms of osteoporosis is secondary to vitamin D shortage; this condition is becoming endemic in western countries because of the reduced exposure to sun and dietary intake [14]; vitamin D optimization (400–1000 IU) and calcium (1500 mg/d) intake is necessary as the use of these supplements determines a significant reduction in the overall number of fractures reaching 15% [13], [15].

VCF by itself is a sign of bone weakness, and it requires a treatment to reverse the overall patient status and to prevent additional fractures from occurring; moreover, drugs acting on bone metabolism can play a role in patients' pain management [16]. Current drugs include bisphosphonates, calcitonin, estrogen, selective estrogen receptor modulators, parathyroid hormone, and receptor activators of nuclear factor kappa-B ligand inhibitor [17]. Bisphosphonates are by far the most prescribed agents, and these are the first line of treatment in symptomatic patients; these act by osteoclast inhibition, leading to reduced bone turnover, increased bone mass, and improved mineralization [18], [19]. Teriparatide shares the first active 34 amino acids of the N-terminal end of the PTH molecule and can increase bone formation when administered intermittently. It reduces back pain, enhances bone mineral density, and decreases the probability of a later fracture; however, it can be prescribed in selected patients, and it cannot be administered in patients with a history of malignancy [20]*.*

Calcitonin was used in the past because of its anabolic impact on bone, and it also provided pain relief through modulating nociception in the central nervous system [9]. The most recent drug for the management of vertebral fractures is Romosozumab, the first anti-sclerostin humanized monoclonal antibody whose effects on bone metabolism and risk of fractures have been recently reported in clinical trials. By binding to sclerostin, permitting the engagement of Wnt ligands with their co-receptors, results in increased bone mineral density (BMD) [21]. Studies confirmed the ability of Romosozumab to decrease the risk of fragility fracture to develop [22], [23].

Pain control is crucial in conservative management. Analgesics are the first line in vertebral compression fracture treatment, generally administrating nonsteroidal anti-inflammatory drugs (NSAIDs). Opioids such as oxycodone can be combined with paracetamol for patients who do not respond well to first-line pain relievers [16]. Among antidepressants, Inhibitors of the Reuptake of Serotonin (SSRIs) can be used short-term in association with standard therapy to control pain in the acute phase in fragile patients; its main drawbacks are the risk of falling at high dosage and light sedation in chronic assumption [24]. Serotonin also plays an important role centrally in functions such as appetite, sleep, sex, and temperature, and recent evidence shows that it may be an important regulatory agent in bone metabolism, increasing bone mass. Furthermore, pregabalin has demonstrated efficacy in pain management, with studies showing a reduction on reducing opioid consumption in the first 24 h post-surgery by 20–62% when used in postoperative analgesia and non-inferior rates of surgery for nonunion [25].

### 2.2 Rest and Brace and Physical Therapy

Soon after a symptomatic VCF, bed rest is advisable because it decreases the axial loads over the fracture site and decreases pain. However, bed rest might result in muscle and bone weakness, pressure sores, and deep vein thrombosis; therefore, as soon as the patients feel better, usually after the first two to four weeks, it is suggested to encourage mobility by wearing a spinal brace. The use of a brace promotes healing by reducing the motion at the fracture site, and it decreases pain by sharing the load and correcting spinal posture. It is deemed that these tools may contrast the kyphotic deformity to develop at the fracture site [26]. When used, a spinal orthosis is recommended in patients with VCF for six to eight weeks approximately. Depending on the location and severity of the fracture, several brace types are available. Thoracolumbar (TLO) brace can be used to treat fractures at the thoracolumbar region, such as the Jewitt, cruciform anterior spinal hyperextension, and the Taylor brace. Thoracolumbar sacral orthoses (TLSO) refer to braces that go all the way to the sacrum, and these are prescribed for fractures at the lumbar vertebral bodies [27]. Braces may also offer different support if rigid, semi-rigid, or soft. Due to its higher discomfort and decreased compliance, the use of rigid bracing has reduced over time [28]. Some patients may benefit from custom made braces to improve wearability, increase the structural support or correct actual deformities as in the case of antigravity braces to correct or prevent kyphotic deformity.

Since braces can determine core muscle weakness and skin complications, patients should be followed up and an appropriate rehabilitation program should follow [29]. Current protocols for the treatment of osteoporosis include at least 30 min treadmill or cycle daily. These training exercises are not possible in patients with acute or subacute VCF because of pain and the increased risk of vertebral body collapse and segmental kyphosis. However, after two months from VCF, return to cycle and physical therapy is appropriate: core strengthening exercises reduce chronic pain, enhance posture and gait, improve the quality of life, and strengthen the back extensors after the initial pain has decreased. Additionally, contrasting the development of sarcopenia, it could decrease the risk of subsequent falls and fractures to occur [16].

### 2.3 Biophysical stimulation

Biophysical stimulation is a noninvasive technique that uses induced electric stimulation to improve bone recovery through the direct and indirect upregulation of bone morphogenic proteins, stimulating bone formation and remodeling. An observational study showed a positive role in osteoblastic function, an inhibition of osteoclast activity, and a clear role in contrasting bone edema, the main characteristic of acute VCFs [30].

**Fig. 4:**
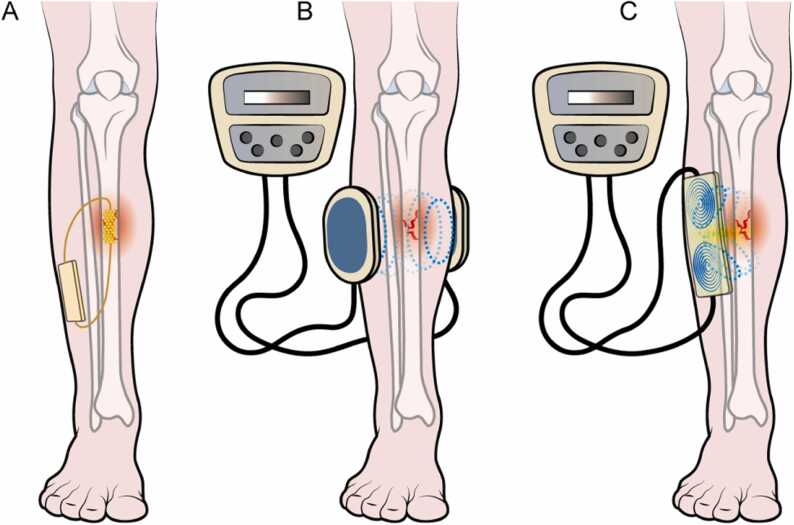
Schematic representation of the three methods of biophysical stimulation: Direct current or DC (A) where a cathode is implanted directly at the fracture site to generate an electric field; Capacitive coupling or CC (B) where two electrodes are located on the two sides of the fracture at skin level and the electric field is generated from an external power source attached to the electrodes; Inductive coupling (IC) where a single electromagnetic current carrying coil is placed on the skin over the fracture site and the electric field is generated from an external power source.

Three methods of biophysical stimulation of osteogenesis have been developed so far, and these include continuous electrical currents directly applied to the bone tissue through implanted electrodes (DC, faradic systems), alternating electrical currents induced externally using pulsed electromagnetic fields in the bone tissue (PEMF, inductive systems), and the alternating electrical currents induced externally using capacitively coupled electric fields (CCEF, capacitive systems) (Fig. 4) [31].

While faradic systems require surgical intervention, albeit minimal, to position the electrodes that release the current at the fracture site, inductive and capacitive systems are non-invasive. The mechanisms of action through which the electrical current applied to the bone tissue with the three methods described above promote osteogenesis are different.

In faradic systems, the direct action of the continuous electrical current manifests with both purely electrical phenomena, which interfere with the dynamics of ions at the fracture site, and chemical processes that lead to a reduction in local oxygen tension and a modest increase in pH. Faradic systems apply higher electrical voltages to the bone tissue compared to inductive or capacitive systems, but their use is solely experimental, and these have not been introduced into clinical practice [32].

The biological activity of PEMF can be explained both through the time-varying magnetic component and the induced electric component, namely the electric field. These signals have complex waveforms, with predominant spectral content ranging from tens of Hertz to tens of thousands of Hertz. The main interaction sites of PEMF are believed to reside at the cell membrane, particularly involving calcium receptors and channels. In vitro experiments have shown that exposure to PEMF promotes the proliferation of human osteoblasts, as well as neoangiogenesis of endothelial cells in cultures. In vivo studies observed an increase in the formation of bone tissue and a reduced time for consolidation of experimental bone fractures. Capacitive systems determine biological effects solely because of the presence of the time-varying electric field. Similar to PEMF these act at the cell membrane and increase osteoblastic activity [33].

In these methods, the waveform of the signal and the intensity, frequency, and duration of the electrical or magnetic stimulation play a crucial role in achieving the desired therapeutic response. The electrical voltage applied can vary between 1 and 10 V, with frequencies ranging from 20 to 200 kHz, and the electric field within the tissue is typically between 1 and 100 mV/cm. The optimal values vary depending on the specific method used [34].

At present, two different types of electrical stimulation are used in clinical settings to promote bone healing: inductive system (PEMF pulsed electromagnetic fields), and capacitive systems (CCEF capacitively coupled electric field) which exploit the properties of the electric field. Recently, a low energy ultrasound-based system to deliver electric fields has been developed and introduced into clinical practice (LIPUS=Low intensity pulsed ultrasound system) [35]. Three biophysical stimulation medical devices produced by IGEA®, EBI® and ORTHOFIX® are available on the market; these non-invasive devices use alternating currents in current-carrying coils on the skin over the fusion site to deliver pulsed electromagnetic field stimulation (PEMF). The pulsed electromagnetic field created by these currents stimulates vascularization, osteoblast migration, an increase in matrix production, and mineralization of developing bone [36]. Because of the morphology of coils and discomfort, for spinal applications, capacitive system is preferred to deliver CCEF at the fracture site [37]. In vitro, in vivo, and clinical studies support the use of PEMF and CCEF to promote bone healing, whose results are reported below.

#### 2.3.1 In vitro studies

**Fig. 5:**
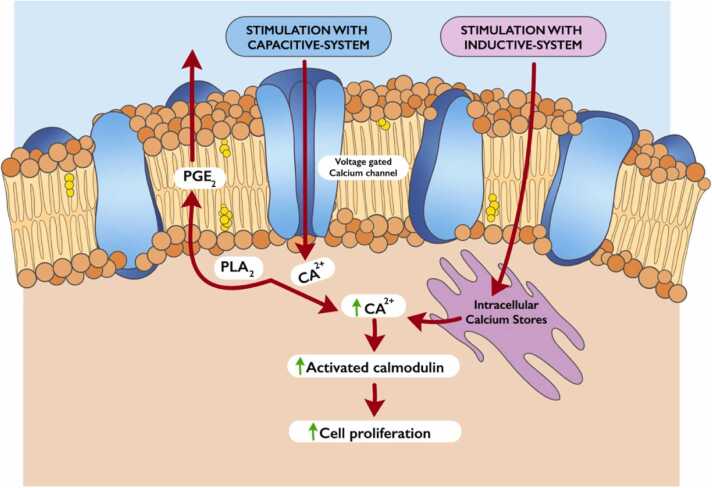
Schematic drawing showing the signal transduction pathways followed by the inductive and capacitive electromagnetic stimulation (PGE_2_ = prostaglandin E_2_, PLA_2_ = phospho- lipase A_2_).

Physical stimulation is recognized and transferred to the various metabolic pathways at the cell membrane level. The inductive system determines the release of calcium ions (Ca++) from the smooth endoplasmic reticulum, while with the capacitive system there is the opening of the membrane channels for the voltage-dependent Ca+ + (Fig. 5).

PEMFs directly control signal transduction by releasing Ca2 + intracellularly, which determines a series of enzymatic reactions resulting in gene transcription and cell proliferation through the synthesis of growth factors such as BMPs, TGF-β and various matrix proteins which lead to an acceleration of the reparation and biomineralization processes [38], [39], [40].

Exposure of bone cell cultures to PEMF stimulates the production and release of growth factors belonging to the TGF-β/BMPs family, which is complemented by a positive anabolic effect, osteogenic differentiation, and increased cell proliferation [41], [42], [43]. It also promotes osteogeneic differentiation of bone marrow and adipose tissue harvested mesenchymal stem cells [44]. It has been observed that electrical stimulation promotes the endogenous synthesis of BMP-2; its effect can be maintained over time because target tissues are exposed for the entire duration of the healing process, and the physical stimulus acts as an endogenous modulator, keeping its activity over time.

The shape of the electromagnetic field is one of the major determinants of cellular effects: continuous low-intensity static magnetic fields (SMF) negatively influence the differentiation and proliferation of osteoblast cell cultures [45] and increase human osteoclast differentiation; on the contrary, pulsed electromagnetic fields (PEMF) minimally impacts osteoclasts differentiation but seem to have an impact on differentiated bone cells reducing osteoclastic resorption and promoting osteoblastic bone deposition [20]. In particular, human osteoblasts isolated from bone tissue samples increase their proliferative activity when exposed to a pulsed electromagnetic field and this effect appears to be higher in osteoblasts isolated from osteoporotic tissue [38], [46].

Other determinants of the final effect include the entity of the electromagnetic field and the duration of the exposure. The first molecular proof of biological effects from 60 kHz EF exposures was provided by Bisceglia et al. who conducted a study on Human liver hepatoma HepG2 cells and Human osteosarcoma SaOS-2 cells [47]. An enhancement in alkaline phosphatase enzymatic activity, a marker of bone regeneration, in both cell lines has also been reported [46]. As regards duration and timing of exposure, Clark et al. stimulated human calvarial osteoblasts with CCEF administrated for 2 h/day and found an up-regulated mRNA expression of TGF-b family genes (BMP-2 and BMP-4, TGF-b1,-b2 and -b3), fibroblast growth factor (FGF)− 2, osteocalcin (BGP) and alkaline phosphatase [48]. Brighton et al. stimulating Cultured MC3T3-E1 bone cells discovered that exposure to capacitive coupling resulted in a significant enhancement in DNA production for any period above thirty minutes by increasing cytosolic Ca2 + , cytoskeletal calmodulin, and prostaglandin E2 all upregulated by the influx of Ca2 + through voltage-gated calcium channels [49].

Pulsed electromagnetic fields (PEMFs) also interact with the adenosine receptors (ARs); therefore, exposure to PEMFs results in a notable increase in the expression of adenosine receptors A2A and A3Ars within different cells or tissues. This increase is associated with a decrease in the levels of several proinflammatory cytokines, particularly A2A and A3Ars receptors exert their anti-inflammatory effect through the inhibition of prostaglandin PGE2 [50]. In vivo tests confirmed the ability of PEMFs to reduce pain and intraarticular inflammation [51].

However, not all forms of electrical stimulation provide the same outcome. Considering direct current (DC), capacitive coupling (CC), pulsed electromagnetic field (PEMF) and degenerate wave (DW), DW showed the greatest proliferative and least apoptotic and cytotoxic effects on cellular activities. More cells invaded collagen as a result of CC and DW, which also produced more MMP-2 and MT1-MMP [52]. (Table 1, Table 2, Table 3).

**Table 1:** In vitro studies.

Author	Year	Cell Cultures	Treatment	Main findings
Bisceglia et al.[47]	2011	Human liver hepatoma HepG2 cells and Human osteosarcoma SaOS-2 cells	Two adhesive planar electro- des placed on the skin paraspinal with 60 kHz EF exposures for 24 h	Increase in alkaline phosphatase (ALP) enzymatic activity in both cell lines: 35% in SaOS-2 cells and 80% in HepG2 cells occurred in the first 4 h after exposure and decreased to almost no change by 24 h.
Brighton et al.[49]	2001	MC3T3-E1 bone cells	Cells exposed to capacitive coupling, stimulation with PEMF, or combined electromagnetic fields at appropriate field strengths for thirty minutes and for two, six, and twenty-four hours	All three signals increased DNA content per dish compared to controls at all time-points, but only exposure to capacitive coupling resulted in a significant ever-increasing DNA production at each time-period beyond thirty minutes.
Caputo et al.[53]	2014	Human SaOS-2 cells	Cells exposed to exposures to a capacitively coupled electrical signal ((60 kHz) with low frequency (LF) for 24 h	No differentially modulated mRNA species Immediately and 4 h after exposure. Differential signals (mRNA encoding transcription factors and DNA binding proteins).
Clark et al.[48]	2014	Human calvarial osteoblasts	Cells grown in modified plastic Cooper dishes and exposed to various capacitively coupled electric fields (60 kHz, 20 mV/ cm, 50% duty cycle) for 2 h per day	Capacitively coupled electric field up-regulated mRNA expression of transforming growth factor (TGF)-b family genes (bone morphogenetic proteins (BMP)− 2 and − 4, TGF-b1, - b2 and -b3), fibroblast growth factor (FGF)− 2, osteocalcin (BGP) and alkaline phosphatae (ALP)
Creecy et al.[42]	2013	Adult human mesenchymal stem cell (MSC)	Cells cultured within electric-conducting type I collagen hydrogels, in the absence of supplemented exogenous dexamethasone and/or growth factors, and exposed to either 10 or 40 mA alternating electric current for 6 h per day	MSCs expressed both early- (such as Runx-2 and osterix) and late- (specifically, osteopontin and osteocalcin) osteogenic genes compared to the control group. Expression of genes pertinent to either adipogenic (specifically, Fatty Acid Binding Protein-4) or chondrogenic (specifically, type II collagen) pathways was not detected when MSCs were exposed to the aforementioned alternating electric-current.
Griffin et al.[52]	2011	Human Bone marrow mesenchymal stem cells (BMMSCs)	Cells exposed to direct current (DC), capacitive coupling (CC), pulsed electromagnetic field (PEMF) and degenerate wave (DW) 3 h per day for 5 days.	DW had the greatest proliferative and least apoptotic and cytotoxic effects. CC and DW caused more cells to invade collagen and showed increased MMP-2 and MT1-MMP expression. DC increased cellular migration and all ES waveforms enhanced expression of migratory genes with DC having the greatest effect.
Hartig et al.[46]	2000	Osteoblast-like primary cells derived from bovine periosteum	Cells exposed to electrical stimulation by capacitively coupled electric fields (16 Hz frequency)	Field application caused acceleration of cell culture development with an enhancement of alkaline phosphatase activity. Exposure of confluent osteoblast-like primary cells to electric fields resulted in enhanced synthesis and secretion of extracellular matrix-related proteins.
Lorich et al.[54]	1998	Rat calvarial bone cells and mouse MC3T3-E1 bone cells	Cells exposed to a capacitively coupled electric field of 20 mV/cm	Field application showed increases in cellular proliferation as determined by deoxyribonucleic acid content. Verapamil, W-7, Indocin and Bromophenacyl bromide inhibited proliferation in cultures subjected to electric field. Neomycin did not inhibit this proliferation.
Wang et al.[43]	2006	Murine cell line MC3T3-E1 cells	Cells exposed to capacitively coupled fields (60 kHz) in which the duration, amplitude, frequency, and duty cycle were sequentially and systematically varied	mRNA levels of BMP-2 through BMP-8, gremlin, and noggin could be significantly up-regulated by specific and selective capacitively coupled electric fields. Concomitantly, BMP-2 protein production and alkaline phosphatase activity were both significantly increased in the same electrically stimulated cultures.
Wiesman et al.[55]	2001	Osteoblast-like cells derived from the periosteal layer of calf metacarpals	Cells exposed to capacitive coupling mode and the semi-capacitive coupling mode of electric pulses for 14 days	Osteoblasts in culture are sensitive to electrical stimulation resulting in an enhancement of the biomineralization process
Xu et al.[40]	2009	Articular chondrocytes isolated from adult bovine patellae	exposed to a capacitively coupled electrical field (60 kHz)	Electrical stimulation involved a pathway of extracellular Ca2þ influx via voltage- gated calcium channels rather than from intracellular Ca2þ repositories; and with downstream roles for calmodulin, calcineurin and nuclear factor of activated T-cells (NF-AT) rather than for phospholipase C and IP3

**Table 2:** In vivo studies.

Author	Year	Models	Treatment	Main findings
Brighton et al.[58]	1983	New Zealand white rabbits	Stimulation with capacitively coupled electrical field at wave signals of 60 kHz frequency and various voltages *(2.5,5,* 10, and 20 V peak-to-peak) at the proximal tibial growth plate for 48 h.	Rabbit growth plate consistently stimulated to statistically significant accelerated growth in a capacitively coupled electrical field. A dose-response effect was noted, with 5 V peak-to-peak exhibiting maximum growth acceleration.
Brighton et al.[56]	1985	New Zealand white rabbits	Right fibula stimulated with capacitively coupled electrical field (60 kHz) continuously for 14 days	Exist a dose- response curve for capacitive coupling and fracture healing. 220 mV, 250 mA, 60 kHz applied electrical signal is the most effective for fracture stimulation in the model studied.
Brighton et al.[59]	1989	Male Sprague Dawley rats	Stimulated with various capacitively coupled electrical fields for six and eight weeks at two and 4.5 months after castration	60 kHz 100 mA signal significantly reversed the castration-induced osteoporosis in the lumbar vertebrae and restored bone mass per unit of volume in rats that had been stimulated for 8 weeks after castration
Carter et al.[60]	1989	Sprague Dawley rats	Stimulated with capacitively coupled electrical field (60 kHz)	Two pair of transversely placed electrodes spaced by at least three vertebral bodies produced the most uniform field distributions. At a current density of 3.0–5.0 mA/cm2 where evidence of a reversal bone loss in castration osteoporosis
Carter et al.[65]	1990	Sprague Dawley rats	Stimulated with capacitively coupled electrical field (60 kHz)	Continuous strip is the best choice of electrodes. The current density generated in cardiac tissue during electrical stimulation at 60 kHz is insufficient to cause cardiac fibrillation. Patients with large amounts of subcutaneous fat require lower input current to maintain the same level of current density in their vertebral bodies as patients with little fat but with the same overall dimensions.
Chan et al.[62]	2019	Sprague Dawley rats	Induced disc generation with percutaneous stab. Rats divided into three groups: sham control, needle stab, needle stab +PEMF. Treated rats exposed to PEMF immediately following surgery and for either 4 or 7 days for 4 h a day.	In untreated animals that at day 7 after injury, inflammatory cytokines and catabolic factors significantly increased at both gene and protein levels. At day 7, PEMF treatment significantly inhibited inflammatory cytokine gene and protein expression induced by needle stab injury. At day 4, PEMF down- regulated FGF-1 and upregulated MMP-2 compared to the stab-only group
Ducheyne et al.[66]	1992	Sprague Dawley rats	Rat tibia stimulated with capacitively coupled electrical field using a porous intramedullary implant	While the current density in the pores are reduced in comparison to the region just outside the pore, a significant current density still exists in the pore region. The presence of the implant increases the current densities in trabecular bone while decreases in cortical bone.
Gilotra et al.[67]	2012	New Zealand white rabbits	Rabbits subjected to a spine infection model with a single dose of intravenously administered systemic ceftriaxone prophylaxis. Rabbits were randomly treated with a capacitive coupling or control device. Instrumentation and soft tissue bacterial growth were assessed after 7 days.	Sites treated with capacitive coupling showed a decrease in the incidence of positive culture: 36% versus 81% in the control group. Overall bacterial load was not decreased with capacitive coupling.
McLeod et al.[61]	1992	Male Turkeys	Left ulnae of turkeys functionally isolated by creation of distal and proximal epiphyseal osteotomies and then exposed to an electrical field for one hour each day for 56 days.	Disuse resulted in a 13% mean loss of osseous tissue. Exposure to the pulsed electrical fields prevents this osteopenia and stimulated a 10 per cent mean increase in the bone area. Osteogenic influence was dependent on the frequency (150, 75 and 15 hertz sinusoidal fields respectively generated a −3%, +5% and +20% mean change in the bone area).
Muttini et al.[37]	2014	Appenninica Breed Sheep	Electricity directly connected with the central pins of an external fixator, stimulated with capacitively coupled electrical field for 12 h daily for 60 days.	Biophysical treatment with alternating electricity in combination with external fixator enhances new-bone formation
Ochi et al.[63]	2003	Japanese White Rabbits	After a dental implant was inserted into each femur of Japanese white rabbits, Solcoseryl (2 ml/kg) was administered intravenously in the ear vein and a capacitively coupled electric field was applied for 4 h per day for 14 days	The degree of bone formation on microscopic observation, bone contact ratio, bone surface area ratio, and the level of removal torque of the implant in the Solcoseryl + CCEF treated group were significantly higher than the control group.
Pepper et al.[64]	1996	Male Beagles	Beagles underwent a right tibia mid-diaphyseal corticotomy, followed by a 5- day delay, and then 21 days of lengthening (1 mm/day). At the start of the post-distraction period (day 27), stimulation (60 kHz) was applied for 28 days.	37% lower maximum torque capacity and a 40% decrease in strain energy to failure in the stimulated group compared with the nonstimulated group. When this dose of capacitive coupled electrical stimulation is applied to the regenerating bone created during distraction osteogenesis, it delays the recovery of bone strength compared with an untreated control.
Yoshida et al.[68]	2009	Male Japanese White Rabbits	Rabbits received external fixation at the right tibia and were assigned to a control group and a fractured group. The bone electrical impedance (Z values) was misured non-invasively by using external fixation pins as electrodes.	Z values in fractured group increased through 5 weeks after surgery while remained constant in control group at 3 weeks. The resistivity and fracture cross.sectional area (FrA) in fractured group decreased through 5 weeks while maximum bending stress (Bmax) increased, reaching a plateau at 5 weeks

**Table 3:** Clinical studies considering vertebral compression fractures and postoperative management after spinal fusion.

Author	Year	Type of Study	Studies included	Study population	Aim	Main findings
Akai et al.[74]	2002	Meta-Analysis	5	180	Evaluate union rate of the fusion site, confirmed with X-ray, and clinical assessment	All studies showed a union of spine fusion confirmed with radio graphic assessment.
Akhter et al.[75]	2020	Systematic Review and Meta-Analysis	7	941	Determine the efficacy of postoperative electrical stimulation on radiographic fusion rates at a minimum 1-year follow-up in adult patients following spinal fusion, analyzing fusion rates relative to smoking status, numbers of levels fused and stimulation method	Electrical stimulation increased the fusion rate by 2.5 times relative to control in non-smokers and 2.8 times relative to control in smokers. The odds of a successful single level fusion were 3 times higher compared to control and 2.6 higher in multi-level fusions. Capacitive coupling had the greatest odds for successful fusion, followed by direct current and pulsed electromagnetic fields
Cottrill et al.[76]	2019	Systematic Review and Meta-Analysis	11 preclinical studies and 13 clinical studies	257 animals and 2144 patients	Overall effect of electrical stimulation technologies on spinal fusion, effect of DCS, ICS and CCS on spinal fusion	Electrical stimulation produced higher rates of fusion compared to control group but with an overall effect smaller than the preclinical studies. DCS and ICS lead to significant decreases in pseudarthrosis rates, whereas CCS does not.
D’Oro et al.[77]	2018	Retrospettive review		2613	Compare the number of patients who underwent a second surgery be- tween those who did and did not receive stimulation.	Among multi- level ALIF+PLF patients, those who underwent stimula- tion exhibited a significantly higher likelihood of revision surgery, null effects of stimulators on the revision rates among the other cohorts. physicians tend to pre- scribe stimulators for more complex and challenging cases (such as multi-level fusion or ALIF+PLF)
Fiani et al.[35]	2021	Review	9		Compare fusion rates of patients undergoing PEMF stimulation	Fusion rates ranged from 64% to 97.6% with PEMF stimulation and 43–86.7% for controls
Gan et Glazer[78]	2006	Review			Summarizes current concepts on the mechanisms of action, animal and clinical studies, and cost justification for the use of electrical stimulation for spinal fusions	DC stimulation to be superior to IC particularly when used to treat posterior spinal fusions. Data on CC therapy also indicate advantages over IC particularly for posterolateral fusions. However, it is not as statistically beneficial as DC for posterior spinal fusions.
Hijji et al.[79]	2018	Meta-analysis	6	924	Compare fusion rates after spinal fusion procedures between patients receiving either electrical stimulation or placebo treatments.	Fusion rates ranged from 35.4% to 90.6% in stimulation groups, and 33.3–92.8% in control groups. There was no significant difference in fusion rates between spinal stimulator and control groups (P = 0.067)
Kahanovitz[80]	2002	Review			Validate the use of various electrical stimulation devices as spinal fusion adjuncts.	Not all adjunctive electrical stimulation is equally effective: direct current is superior to PEMF particularly when used to enhance posterior spinal fusions. Also capacitive coupling shows clinical superiority over PEMF.
Oishi et Onest[81]	2000	Review	8		Provide the indications and limitations of electrical stimulation to enhance spinal fusion	Evidence supports its use for selected indications: multilevel fusion, reoperation for pseudoarthrosis and the presence of osteoporosis, smoking or significant vascular disease.
Tian et al.[82]	2013	Meta-analysis	21	1381	Determine Fusion rates using radiography or computed tomography.	No statistically significant differences among the three electrical stimulation methods with an overall fusion efficacy of 85%

#### 2.3.2 In vivo studies

Since 1983, numerous studies have been conducted to test the efficacy of biophysical stimulation in animal models. First, Brighton et al. demonstrated on Sprague-Dawley rat model that capacitive system can prevent or reverse disuse osteoporosis in the rat limb with a dose-response effect [49], [56], [57], [58]. They also showed how 8 weeks of stimulation with CCEF reversed the castration-induced osteoporosis in the lumbar vertebrae and restored bone mass per unit of volume in rats models [59]. Similar findings have been observed by Carter et al. [60] and McLeod et al. [61] which found a10 per cent mean increase in the bone area stimulated with CCEF and a reduction of osteopenia, particularly expressed on the frequency of 15 hertz. The anti-inflammatory effect of PEMFs was also studied in Sprague Dawley Rats models, proving their inhibitory effect on acute inflammatory cytokine expression, resulting from the downregulation of FGF-1 and upregulating MMP-2 compared to the control group [62]. In addition, the effects of electrical stimulation were also evaluated when administrated along with a tissue respiration stimulating agent (Solcoseryl) through the study of dental implants and their osseointegration into the surrounding bone. In this context, an increased levels of bone formation on microscopic observation, bone contact ratio, bone surface area ratio, and the level of removal torque of the implant have been documented [63]. Most recently. Muttini et. al. [37] used an appenninica breed sheep model to study CCEF stimulation for fracture healing, showing that an external fixator used in addition to biophysical stimulation with alternating electricity may accelerate the rate of callus maturation. However, in contrast with the proven utility of CCEF in healing delayed or nonunions, its use in treating regenerating bone from distraction osteogenesis appears to be contraindicated, showing a reduction in the rate of recovery of torsional strength and a subsequent delay in bone strength restoration [64].

#### 2.3.3 Clinical studies

Clinicians successfully employed biophysical stimulation to support reparative osteogenesis, and post-traumatic fracture healing in VCFs [69]. Clinical studies showed the beneficial impact on pain through a decreased requirement of pain medications. CCEFs have the theoretical advantage, compared to pharmacological administration, of producing locally a constant increase in the concentration of growth factors without the use of large initial dosages, which may accompany local or systemic toxic effects. Rossini et al. [70], in a study conducted on 65 postmenopausal women with radiographically documented multiple vertebral osteoporotic fractures at the thoracolumbar level and chronic pain unsuccessfully treated with NSAIDs for at least 6 months demonstrated that CCEF stimulation was successful in promoting the healing of spinal fusion; moreover, a positive effect on pain without causing adverse events after prolonged use was observed. Patients were treated by Osteospine® (IGEA SpA Carpi (Mo); Italy) for a minimum of 9 h per day for 2 months. The same beneficial effect was demonstrated in patients treated after lumbar spine fusion surgery [71], [72], [73]. In another study, Piazzolla et al. considered 24 patients with acute VCFs, dividing them into two groups conservatively treated with or without CCEF. Patients were managed by Osteospine® (IGEA SpA Carpi (Mo); Italy) for 8 h a day for 3 months. At 90 day follow-up, patients treated by CCEF showed a higher improvement of clinical symptoms and faster fracture healing and BME resolution [30].

## 3 Orthopedic surgery

When conservative treatment is unsuccessful, surgery can be required. Surgical management of VCF is rare, and patients and surgeons should be aware of the risk to benefit ratio in this peculiar patients population. When surgery is required, it includes cementoplasty procedures, either vertebroplasty or kyphoplasty, or instrumented spinal fixation [83]. In individuals with burst fractures, cementoplasty significantly increases the height of the anterior column, thereby reducing local kyphosis, and reducing pain [84]; despite it is rarely associated with complications, it may promote fractures at the adjacent vertebral bodies [85]. Segmental fusion aims for segmental alignment correction; however, it increases the stress on adjacent segments and, in patients with poor bone quality, implant loosening with segmental kyphosis may occur. In case of major spinal imbalance or neurological deficits, open reduction and spinal fusion are required [86] [87].

In 2016, the North American Spine Society (NASS) issued recommendations regarding the use of electrical stimulation for bone healing, as an aid for spinal fusion. These recommendations highlighted specific clinical scenarios and qualifying criteria for the use of electrical stimulation in the different regions of the spine, including the occipital-cervical, cervical, cervicothoracic, thoracic, thoracolumbar, lumbar, and lumbosacral regions. The guidelines emphasized that electrical stimulation can be considered as an adjunctive therapy for spinal fusion in patients at high risk of developing nonunion. Additionally, the guidelines mentioned the potential utilization of non-invasive electrical stimulation in patients with delayed union in lumbar fusion, either with or without associated risk factors [88].

## 4 Conclusions

VCFs are the clinical manifestation most frequently associated with a weakened bone. The formation of a less resistant bone callus, delayed functional recovery, increased risk of subsequent fractures, mobilization of artificial devices and poor osseointegration of prosthetic implants are just a few of the direct or treatment-related complications of fragility fractures. Optimal care in patients with VCF should include the etiological assessment of osteoporosis to rule out secondary causes [17], and most patients benefit from MM. In this setting, biophysical stimulation contributes to the effectiveness of MM of VCFs, promoting accelerated healing, and supporting the reduction of bed rest and pain medications. Economic cost-effective analysis in the long term is required to support their widespread use in clinical practice [36] [81].

In patients with VCF, integrated MM is the gold standard of treatment, consisting of a combination of brace, pain management, bone metabolism evaluation, osteoporosis medication and biophysical stimulation; this treatment improves patients’ quality of life, promotes healing, and reduces the risk to undergo surgery in patients affected by VCFs [70], [89], [73].

Limitations include the possibility of inducing oxidative stress, because of the increased blood flow and circulation carried on by nitric oxide emission which leads to oxygen radicals accumulation and the risk of lowering blood pressure and decreasing heart rate while on PEMF therapy [35],; another limitation is the overall increased costs, not necessarily supported by an improved outcome [79].

Future perspectives may involve the implementation of the computational engineering approach to determine the interaction of different magnetic fields shape and amplitude in vertebrae and other bones with and without the presence of bone cement or instrumentation. Moreover, it could be useful at a cellular level to understand the patterns and predict the rate of resolution of bone edema in fractured vertebras. Finally, it could be useful to improve current devices in the directions of patient specific therapies by matching specific bone edema patterns and fracture patterns to specific deliverable magnetic field amplitudes and shapes [90].

## Ethics approval

no ethical approval is required.

## Compliance with ethical standards

The authors declare that they have no conflict of interest.

## Funding

The authors declare that no funds, grants, or other support were received during the preparation of this manuscript.

## CRediT authorship contribution statement

The authors assert that this manuscript is original, has not been previously published, and is not presently under consideration for publication elsewhere. We affirm that all named authors have read and approved the manuscript, and there are no other individuals who meet the criteria for authorship but are not included. Additionally, we confirm unanimous agreement on the order in which authors are listed in the manuscript.

## CRediT authorship contribution statement

All authors contributed to the study conception and design. All authors contributed to the study conception and design. Material preparation, data collection and analysis were performed by Alberto Di Martino, Eleonora Villari, Riccardo Poluzzi, Matteo Brunello, Valentino Rossomando, Claudio D’Agostino, Federico Ruta e Cesare Faldini. The first draft of the manuscript was written by Alberto Di Martino and all authors commented on previous versions of the manuscript.

## Declaration of Competing Interest

The authors have no relevant financial or non-financial interests to disclose.

## References

[1] Sànchez-Riera L., Wilson N. (2017). Fragility fractures & their impact on older people. Best Pr Res Clin Rheuma.

[2] Pisani P., Renna M.D., Conversano F. (2016). Major osteoporotic fragility fractures: risk factor updates and societal impact. World J Orthop.

[3] Noel S.E., Santos M.P., Wright N.C. (2021). Racial and ethnic disparities in bone health and outcomes in the United States. J Bone Min Res.

[4] Johnell O., Kanis J. (2005). Epidemiology of osteoporotic fractures. Osteoporos Int.

[5] Kado D.M., Browner W.S., Palermo L. (1999). Vertebral fractures and mortality in older women: a prospective study. Study of Osteoporotic Fractures Research Group. Arch Intern Med.

[6] Joestl J., Lang N., Bukaty A. (2017). Osteoporosis associated vertebral fractures—health economic implications. PLOS ONE.

[7] Prather H., Hunt D., Watson J.O., Gilula L.A. (2007). Conservative care for patients with osteoporotic vertebral compression fractures. Phys Med Rehabil Clin N Am.

[8] Karaca L., Yuceler Z., Kantarci M. (2016). The feasibility of dual-energy CT in differentiation of vertebral compression fractures. Br J Radio.

[9] Patel D., Liu J., Ebraheim N.A. (2022). Managements of osteoporotic vertebral compression fractures: a narrative review. World J Orthop.

[10] Prost S., Pesenti S., Fuentes S. (2021). Treatment of osteoporotic vertebral fractures. Orthop Trauma: Surg Res.

[11] Barr J.D., Jensen M.E., Hirsch J.A. (2014). Position statement on percutaneous vertebral augmentation: a consensus statement developed by the Society of Interventional Radiology (SIR), American Association of Neurological Surgeons (AANS) and the Congress of Neurological Surgeons (CNS), American College of Radiology (ACR), American Society of Neuroradiology (ASNR), American Society of Spine Radiology (ASSR), Canadian Interventional Radiology Association (CIRA), and the Society of NeuroInterventional Surgery (SNIS). J Vasc Inter Radio.

[12] Aras E.L., Bunger C., Hansen E.S., Søgaard R. (2016). Cost-effectiveness of surgical versus conservative treatment for thoracolumbar burst fractures. Spine.

[13] Stein E., Shane E. (2003). Secondary osteoporosis. Endocrinol Metab Clin North Am.

[14] Lips P., Cashman K.D., Lamberg-Allardt C. (2019). Current vitamin D status in European and Middle East countries and strategies to prevent vitamin D deficiency: a position statement of the European Calcified Tissue Society. Eur J Endocrinol.

[15] Weaver C.M., Alexander D.D., Boushey C.J. (2016). Calcium plus vitamin D supplementation and risk of fractures: an updated meta-analysis from the National Osteoporosis Foundation. Osteoporos Int.

[16] Jang H.-D., Kim E.-H., Lee J.C. (2022). Management of osteoporotic vertebral fracture: review update 2022. Asian Spine J.

[17] Cosman F., de Beur S.J., LeBoff M.S. (2014). Clinician’s guide to prevention and treatment of osteoporosis. Osteoporos Int.

[18] Reszka A.A., Rodan G.A. (2003). Bisphosphonate mechanism of action. Curr Rheuma Rep.

[19] Deeks E.D. (2018). Denosumab: a review in postmenopausal osteoporosis. Drugs Aging.

[20] Anagnostis P., Gkekas N.K., Potoupnis M. (2019). New therapeutic targets for osteoporosis. Maturitas.

[21] Em L.B., Jp B L. (2017). Romosozumab for the treatment of osteoporosis. Expert Opin Biol Ther.

[22] Fixen C., Tunoa J. (2021). Romosozumab: a review of efficacy, safety, and cardiovascular risk. Curr Osteoporos Rep.

[23] Kg S., J P., Ml B. (2017). Romosozumab or alendronate for fracture prevention in women with osteoporosis. N Engl J Med.

[24] Rizzoli R., Cooper C., Reginster J.-Y. (2012). Antidepressant medications and osteoporosis. Bone.

[25] Castillo R.C., Raja S.N., Frey K.P. (2017). Improving pain management and long-term outcomes following high-energy orthopaedic trauma (pain study). J Orthop Trauma.

[26] Jin Y.Z., Lee J.H. (2016). Effect of brace to osteoporotic vertebral fracture: a meta-analysis. J Korean Med Sci.

[27] Wong C.C., McGirt M.J. (2013). Vertebral compression fractures: a review of current management and multimodal therapy. J Multidiscip Health 6.

[28] Slavici A., Rauschmann M., Fleege C. (2017). Conservative management of osteoporotic vertebral fractures: an update. Eur J Trauma Emerg Surg.

[29] McCARTHY J., Davis A. (2016). Diagnosis and management of vertebral compression fractures. afp.

[30] Piazzolla A., Solarino G., Bizzoca D. (2015). Capacitive coupling electric fields in the treatment of vertebral compression fractures. J Biol Regul Homeost Agents.

[31] Haupt H.A. (1984). Electrical stimulation of osteogenesis. South Med J.

[32] Khaw J.S., Xue R., Cassidy N.J., Cartmell S.H. (2022). Electrical stimulation of titanium to promote stem cell orientation, elongation and osteogenesis. Acta Biomater.

[33] Shankar V.S., Simon B.J., Bax C.M. (1998). Effects of electromagnetic stimulation on the functional responsiveness of isolated rat osteoclasts. J Cell Physiol.

[34] The regenerative effects of electromagnetic field on spinal cord injury: Electromagnetic Biology and Medicine: Vol 36, No 1. https://www.tandfonline.com/doi/full/10.3109/15368378.2016.1160408. Accessed 13 Aug 2023. 10.3109/15368378.2016.1160408 27398987

[35] Fiani B., Kondilis A., Runnels J. (2021). Pulsed electromagnetic field stimulators efficacy for noninvasive bone growth in spine surgery. J Korean Neurosurg Soc.

[36] Wade S.M., Clark D.M., Fredericks D.R., Wagner S.C. (2020). Pulsed electromagnetic field stimulation is a practical adjunctive therapy for fusion in spine surgery. Clin Spine Surg.

[37] Muttini A., Abate M., Bernabò N. (2014). Effect of electric current stimulation in combination with external fixator on bone healing in a sheep fracture model. Vet Ital.

[38] Sollazzo V., Palmieri A., Pezzetti F. (2010). Effects of pulsed electromagnetic fields on human osteoblastlike cells (MG-63): a pilot study. Clin Orthop Relat Res.

[39] Wiesmann H.-P., Hartig M., Stratmann U., et al Electrical stimulation in£uences mineral formation of osteoblast-like cells in vitro. Biochimica et Biophysica Acta. 10.1016/s0167-4889(00)00135-x 11341980

[40] Xu J., Wang W., Clark C.C., Brighton C.T. (2009). Signal transduction in electrically stimulated articular chondrocytes involves translocation of extracellular calcium through voltage-gated channels. Osteoarthr Cartil.

[41] Massari L., Benazzo F., Falez F. (2019). Biophysical stimulation of bone and cartilage: state of the art and future perspectives. Int Orthop.

[42] Creecy C.M., O’Neill C.F., Arulanandam B.P. (2013). Mesenchymal stem cell osteodifferentiation in response to alternating electric current. Tissue Eng Part A.

[43] Wang Z., Clark C.C., Brighton C.T. (2006). Up-regulation of bone morphogenetic proteins in cultured murine bone cells with use of specific electric fields. J Bone Jt Surg Am.

[44] Ongaro A., Pellati A., Bagheri L. (2014). Pulsed electromagnetic fields stimulate osteogenic differentiation in human bone marrow and adipose tissue derived mesenchymal stem cells. Bioelectromagnetics.

[45] Denaro V., Cittadini A., Barnaba S.A. (2008). Static electromagnetic fields generated by corrosion currents inhibit human osteoblast differentiation. Spine (Philos Pa 1976).

[46] Hartig M., Joos U., Wiesmann H.P. (2000). Capacitively coupled electric fields accelerate proliferation of osteoblast-like primary cells and increase bone extracellular matrix formation in vitro. Eur Biophys J.

[47] Bisceglia B., Zirpoli H., Caputo M. (2011). Induction of alkaline phosphatase activity by exposure of human cell lines to a low-frequency electric field from apparatuses used in clinical therapies. Bioelectromagnetics.

[48] Clark C.C., Wang W., Brighton C.T. (2014). Up-regulation of expression of selected genes in human bone cells with specific capacitively coupled electric fields. J Orthop Res.

[49] Brighton C.T., Wang W., Seldes R. (2001). Signal transduction in electrically stimulated bone cells. J Bone Jt Surg Am.

[50] Varani K., Vincenzi F., Ravani A. (2017). Adenosine Receptors as a Biological Pathway for the Anti-Inflammatory and Beneficial Effects of Low Frequency Low Energy Pulsed Electromagnetic Fields. Mediat Inflamm.

[51] Moretti L., Bizzoca D., Geronimo A. (2023). Targeting adenosine signalling in knee chondropathy: the combined action of polydeoxyribonucleotide and pulsed electromagnetic fields: a current concept review. Int J Mol Sci.

[52] Griffin M., Iqbal S.A., Sebastian A. (2011). Degenerate wave and capacitive coupling increase human msc invasion and proliferation while reducing cytotoxicity in an in vitro wound healing model. PLOS ONE.

[53] Caputo M., Zirpoli H., De Rosa M.C. (2014). Effect of low frequency (LF) electric fields on gene expression of a bone human cell line. Electro Biol Med.

[54] Lorich D.G., Brighton C.T., Gupta R. (1998). Biochemical pathway mediating the response of bone cells to capacitive coupling. Clin Orthop Relat Res.

[55] Wiesmann H., Hartig M., Stratmann U. (2001). Electrical stimulation influences mineral formation of osteoblast-like cells in vitro. Biochim Biophys Acta.

[56] Brighton C.T., Hozack W.J., Brager M.D. (1985). Fracture healing in the rabbit fibula when subjected to various capacitively coupled electrical fields. J Orthop Res.

[57] Brighton C.T., Wang W., Clark C.C. (2008). The effect of electrical fields on gene and protein expression in human osteoarthritic cartilage explants. J Bone Jt Surg Am.

[58] Brighton C.T., Pfeffer G.B., Pollack S.R. (1983). In vivo growth plate stimulation in various capacitively coupled electrical fields. J Orthop Res.

[59] Brighton C.T., Luessenhop C.P., Pollack S.R. (1989). Treatment of castration-induced osteoporosis by a capacitively coupled electrical signal in rat vertebrae.. J Bone Jt Surg Am.

[60] Carter E.L., Vresilovic E.J., Pollack S.R., Brighton C.T. (1989). Field distributions in vertebral bodies of the rat during electrical stimulation: a parametric study.. IEEE Trans Biomed Eng.

[61] McLeod K.J., Rubin C.T. (1992). The effect of low-frequency electrical fields on osteogenesis.. J Bone Jt Surg Am.

[62] Chan A.K., Tang X., Mummaneni N.V. (2019). Pulsed electromagnetic fields reduce acute inflammation in the injured rat-tail intervertebral disc. JOR Spine.

[63] Ochi M., Wang P.-L., Ohura K. (2003). Solcoseryl, a tissue respiration stimulating agent, significantly enhances the effect of capacitively coupled electric field on the promotion of bone formation around dental implants. Clin Oral Implants Res.

[64] Pepper J.R., Herbert M.A., Anderson J.R., Bobechko W.P. (1996). Effect of capacitive coupled electrical stimulation on regenerate bone. J Orthop Res.

[65] Carter E.L., Pollack S.R., Brighton C.T. (1990). Theoretical determination of the current density distributions in human vertebral bodies during electrical stimulation. IEEE Trans Biomed Eng.

[66] Ducheyne P., Ellis L.Y., Pollack S.R. (1992). Field distributions in the rat tibia with and without a porous implant during electrical stimulation: a parametric modeling. IEEE Trans Biomed Eng.

[67] Gilotra M., Griffith C., Schiavone J. (2012). Capacitive coupling reduces instrumentation-related infection in rabbit spines: a pilot study. Clin Orthop Relat Res.

[68] Yoshida T., Kim W.-C., Kawamoto K. (2009). Measurement of bone electrical impedance in fracture healing. J Orthop Sci.

[69] Nelson F.R.T., Brighton C.T., Ryaby J. (2003). Use of physical forces in bone healing. J Am Acad Orthop Surg.

[70] Rossini M., Viapiana O., Gatti D. (2010). Capacitively coupled electric field for pain relief in patients with vertebral fractures and chronic pain. Clin Orthop Relat Res.

[71] Massari L., Brodano G.B., Setti S. (2020). Does capacitively coupled electric fields stimulation improve clinical outcomes after instrumented spinal fusion? A multicentered randomized, prospective, double-blind, placebo-controlled trial. Int J Spine Surg.

[72] Goodwin C.B., Brighton C.T., Guyer R.D. (1999). A double-blind study of capacitively coupled electrical stimulation as an adjunct to lumbar spinal fusions. Spine.

[73] Massari L. (2011). Algorithm for employing physical forces in metabolic bone diseases. Aging Clin Exp Res.

[74] Akai M., Kawashima N., Kimura T., Hayashi K. (2002). Electrical stimulation as an adjunct to spinal fusion: a meta-analysis of controlled clinical trials. Bioelectromagnetics.

[75] Akhter S., Qureshi A.R., Aleem I. (2020). Efficacy of electrical stimulation for spinal fusion: a systematic review and meta-analysis of randomized controlled trials. Sci Rep.

[76] Cottrill E., Pennington Z., Ahmed A.K. (2019). The effect of electrical stimulation therapies on spinal fusion: a cross-disciplinary systematic review and meta-analysis of the preclinical and clinical data. J Neurosurg Spine.

[77] D’Oro A., Buser Z., Brodke D.S. (2018). Trends and costs of external electrical bone stimulators and grafting materials in anterior lumbar interbody fusion. Asian Spine J.

[78] Gan J.C., Glazer P.A. (2006). Electrical stimulation therapies for spinal fusions: current concepts. Eur Spine J.

[79] Hijji F.Y., Narain A.S., Haws B.E. (2018). The efficacy of electrical spinal fusion stimulators on fusion rates: a meta-analysis. Curr Orthop Pract.

[80] Kahanovitz N. (2002). Electrical stimulation of spinal fusion: a scientific and clinical update. Spine J.

[81] Oishi M., Onesti S.T. (2000). Electrical bone graft stimulation for spinal fusion: a review. Neurosurgery.

[82] Tian N.F., Wu Y.S., Zhang X.L. (2013). Efficacy of electrical stimulation for spinal fusion: a meta-analysis of fusion rate. Spine J.

[83] Petitt J.C., Desai A., Kashkoush A. (2022). Failure of conservatively managed traumatic vertebral compression fractures: a systematic review. World Neurosurg.

[84] Hoffmann J., Preston G., Whaley J., Khalil J.G. (2023). Vertebral augmentation in spine surgery. J Am Acad Orthop Surg.

[85] Park J.-S., Park Y.-S. (2021). Survival analysis and risk factors of new vertebral fracture after vertebroplasty for osteoporotic vertebral compression fracture. Spine J.

[86] Robinson Y., Heyde C.E., Försth P., Olerud C. (2011). Kyphoplasty in osteoporotic vertebral compression fractures - guidelines and technical considerations. J Orthop Surg Res.

[87] Alpantaki K., Dohm M., Korovessis P., Hadjipavlou A.G. (2018). Surgical options for osteoporotic vertebral compression fractures complicated with spinal deformity and neurologic deficit. Injury.

[88] (PDF) Nass coverage policy recommendations Cervical Artificial Disc Replacement | Khooshund Ramlugon - Academia.edu. 〈https://www.academia.edu/40104090/NASS_COVERAGE_POLICY_RECOMMENDATIONS_Cervical_Artificial_Disc_Replacement〉. Accessed 8 Sep 2023.

[89] Liu W., Jin X., Guan Z., Zhou Q. (2021). Pulsed electromagnetic field affects the development of postmenopausal osteoporotic women with vertebral fractures. Biomed Res Int.

[90] Markov M.S. (2007). Pulsed electromagnetic field therapy history, state of the art and future. Environmentalist.

